# Enhancing health security in Africa: Collaboration between public health institutes and academia

**DOI:** 10.4102/jphia.v16i1.780

**Published:** 2025-04-16

**Authors:** Andrew Kambugu, Rodgers R. Ayebare, Francis Kakooza, Agnes Kiragga, Aster Tsegaye, Georgina Yeboah, Leah Mbabazi, Tonny Muwonge, Tamrat Shaweno, Nebiyu Dereje, Elizabeth Gonese, Mosoka P. Fallah, Tajudeen Raji, Ngashi Ngongo, Jean Kaseya

**Affiliations:** 1 Infectious Diseases Institute, Makerere University, Kampala, Uganda; 2 Global Health Security Programme, Infectious Diseases Institute, Makerere University, Kampala, Uganda; 3 African Population and Health Research Centre, Nairobi, Kenya; 4 Africa Forum for Research and Education in Health, Kumasi, Ghana; 5 College of Health Sciences, University of Addis Ababa, Addis Ababa, Ethiopia; 6 Africa Centers for Disease Control and Prevention, Addis Ababa, Ethiopia

**Keywords:** COVID-19, vaccines, collaboration, Africa health security, evidence-based public health, saving lives and livelihoods, research to action, health systems

## Abstract

**Background:**

The COVID-19 pandemic exposed significant challenges in Africa, including weak health systems, misinformation, limited vaccine access, and a lack of local data. Effective country-level leadership, coordination, and communication were crucial in addressing these multifaceted challenges.

**Aim:**

The Program for Research on Vaccine Effectiveness (PROVE) was established to address these challenges by fostering international partnerships and advancing scientific knowledge generation toward implementing the Africa Centers for Disease Control and Prevention’s New Public Health Order.

**Setting:**

The PROVE program is a collaborative initiative under the Saving Lives and Livelihoods initiative of Africa CDC and the Mastercard Foundation. It brings together a diverse network of researchers and policymakers from National Public Health Institutions and academia across Africa.

**Methods:**

The programme employs a variety of qualitative and quantitative research methods, including surveys, interviews, focus groups, and analysis of existing data, along with capacity-building efforts.

**Results:**

The PROVE program has achieved several significant results, including: developing evidence-based policy recommendations to strengthen DHIS2 and other data systems; strengthening the capacity of National Public Health Institutions; fostering collaboration among researchers, policymakers, and public health professionals; and promoting the use of local data for evidence-informed decision-making.

**Conclusion:**

The PROVE programme’s collaborative approach is a valuable initiative with the potential to improve evidence generation and utilisation in Africa. By encouraging collaboration, knowledge generation and building capacity of local scientists in implementation science, the initiative helps address the challenges posed by the COVID-19 pandemic and builds resilience against future public health emergencies.

**Contribution:**

This paper describes PROVE’s innovative approaches to generating new knowledge, developing practical approaches, and building capacity among African researchers in collaboration with policymakers.

## Introduction

The symbiotic relationship between academia and policymakers has long been recognised as crucial for evidence-based decision-making in public health.^1^ However, a persistent gap between these two spheres has impeded the effective translation of research into practical policies, resulting in missed opportunities to enhance public health practices.^2^ The emergence of the COVID-19 pandemic further highlighted the ramifications of this gap, underscoring the dire need for seamless collaboration between academia and policymakers.^3^

One of the most glaring revelations during the pandemic was the mismatch between the pace of the research process and the rapid evolution of the resultant public health crises. As the virus spread across the globe, researchers scrambled to provide timely insights, often outpaced by the urgency of policy decisions. This dynamic exacerbated the existing divide between the evidence-generation process and its application in shaping public health interventions.^4^

To bridge this gap and create a more effective ecosystem of evidence-based public health practice, a novel approach has gained traction: Ministries of Health working through their respective National Public Health Institutes and academia to co-lead policy-informing research.

The Program for Research on Vaccine Effectiveness (PROVE) in Africa, an initiative under the Saving Lives and Livelihoods initiative, is championing this approach through research and capacity building, primarily working with NPHIs.

This approach seeks to harmonise the strengths of both academia and policymakers, emphasising the importance of local contexts and evidence in guiding decision-making. By involving academia as evidence custodians, policy recommendations can be informed by robust research outcomes grounded in the local realities of health systems, social dynamics, and cultural nuances.^5,6,7,8^

Furthermore, the programme provides a practical example of implementing the New Public Health Order for Africa, a strategy to ensure sustained health outcomes and health security. PROVE directly supports the strengthening of public health institutions, the development of the public health workforce, and the cultivation of respectful, action-oriented partnerships. Thus, it works in alignment with the New Public Health Order for Africa, with a significant focus in expanding the local manufacturing of vaccines, diagnostics, and therapeutics.

## Reseach methods and design

### The saving lives and livelihoods initiative

The PROVE program is part of the Implementation Science pillar of the ‘Saving Lives and Livelihoods’ initiative of the Africa Centres for Disease Control and Prevention (Africa CDC) and the Mastercard Foundation, which aims to rapidly expand COVID-19 vaccination across Africa.^9^

This pillar is mandated to use a research and capacity-building approach to assess the real-world effectiveness of COVID-19 vaccines, the impact of the pandemic on essential health service delivery, and identify barriers and successful strategies for practical vaccination efforts.^10^

### Partnerships and stakeholders engagement strategy

The PROVE program is based on respectful and action-oriented partnerships to achieve mutual benefits for African Union (AU) member states, Africa CDC, the Mastercard Foundation, and the implementing partners. Africa CDC determined priority research areas. Knowledge gaps in implementing a vaccination programme on the continent informed the priorities. The PROVE program used the research approach to answer the following priority questions pertaining to the continental response to the pandemic: What is the real-world effectiveness of COVID-19 vaccines in Africa?; What factors hinder or facilitate the uptake of COVID-19 vaccines among healthcare workers?; What is the pandemic’s impact on Africa’s health systems?

Africa CDC, as the sponsor of these research endeavours, played a pivotal role in connecting the implementing partners’ consortium with AU member states. Furthermore, Africa CDC was responsible for reviewing and approving the research protocol, aiding research teams in accessing health data from member states, and providing research data management services. The Mastercard Foundation allocated resources to the programme and continues to support the monitoring and evaluation of its implementation.

The Makerere University, Infectious Diseases Institute (IDI) partnered with the Africa Forum for Research and Education in Health (AFREhealth) to lead the PROVE consortium. AFREhealth was a strategic partner with a continental presence that identified and seconded experienced faculty based in local universities and research organisations to support programme implementation in the respective member states.

National Public Health Institutes (NPHIs) are scientific entities that coordinate public responses and are accountable to the MoH. A core mandate of the NPHIs is conducting research and producing data to shape public health policies and interventions. Within the PROVE framework, directors or their designated representatives serve as principal investigators (PIs) of the initiative. This approach has yielded two key advantages: fostering country-level ownership to ensure the effective use of findings and aligning research efforts with national strategies for controlling COVID-19. AFREhealth-affiliated scientists were designated as co-principal investigators (Co-PIs). Their role has been to provide specialised technical support in implementing the research protocols related to COVID-19, leveraging their expertise to contribute to the programme’s success.

### Identification of priority research areas and research sites

In consultation with Africa CDC, the PROVE consortium conducted an objective study site selection process. This was guided by key principles, including representativeness, the strength of existing COVID-19 surveillance programmes, and the public health relevance of the research questions across AU member state. The team utilised the World Health Organization guidance on conducting vaccine effectiveness evaluations to shape the country selection strategies.^11^ This guidance outlines 10 criteria, summarised in Table 1.^11^

**TABLE 1:** Adoption of suggested criteria to undertake vaccine effectiveness evaluations of COVID-19 vaccines under the PROVE program.

Definition of a criterion	Adoption for assessment of AU member states
Clear public health rationale	The team appraised the prevailing COVID-19 vaccination coverages from the Africa CDC website. Countries were categorised into three tiers of national COVID-19 coverage, i.e., less than 10%, 10% – 60%, and more than 60%. Countries with lower vaccination coverage were given priority.
Experienced epidemiologic team	A scientist with a track record of conducting public health research affiliated with a local research institution was identified and vetted through the AFREhealth network.
Dedicated staffing	Sites that had functional National Institutes of Public Health or sites that were in the process of establishing one were prioritised.
Identified sites of enrolment	We reviewed the available country support plans and prioritised AU member states that had requested COVID-19 vaccination centres and pharmacovigilance support.
Availability of reliable diagnostic tests in the study population	By reviewing country support plans, AU member states that had requested support to conduct genomic surveillance were prioritised as having the minimum capacity to conduct nucleic acid-based tests for SARS-CoV2. However, this criterion was dropped due to the widespread adoption of nucleic acid-based tests as indicated on the Africa CDC-PANABIOS consortium Trusted Travel portal.^20^
Ability to ascertain the vaccination status accurately	At the planning stage, we considered the availability of local vaccination uptake dashboards on the Ministry of Health websites as a proxy for the existence of national databases for COVID-19 vaccination. Later, at the microplanning stage, the team further appraised the verification of vaccination status by reviewing information on national vaccination databases and evidence given to individuals after vaccination.
Data collection, management, and analytic capacity in place	Like how we ascertained vaccination status, the online availability of national COVID-19 vaccination dashboards was considered a proxy for this capability. We further augmented this assessment by appraising participation in previous African CDC-led surveillance efforts, such as the Partnership for Evidence-Based Response (PERC) to COVID-19.
Ability to enrol enough participants	This criterion was pre-emptively fulfilled by adopting a dual research method for vaccine effectiveness. The primary approach was to evaluate effectiveness using a retrospective study methodology, with the flexibility to recruit prospectively in the event of a new surge in SARS-CoV-2 cases.
Data dissemination plan in place	AU member states that actively published COVID-19 surveillance reports on the Ministry of Health websites were prioritised. Dissemination through the Africa CDC communication channels was also considered a backup to this capability.
Funding secured to conduct rigorous evaluation	This criterion was fulfilled a priori by participation in the SLL initiative.
A functional ethical review committee	The team reviewed existing literature on the availability of ethics committees in Africa,^21,22^ and AU member states with practical ethics committees were prioritised for participation in this programme.

*Source*: Adapted from WHO. Evaluation of COVID-19 vaccine effectiveness [Internet]. 2021 [cited 2023 Nov 6]. Available from: https://www.who.int/publications/i/item/WHO-2019-nCoV-vaccine_effectiveness-measurement-2021.1

CDC, Centres for Disease Control and Prevention; PROVE, Program for Research on Vaccine Effectiveness; AU, African Union; SLL, Saving Lives and Livelihoods.

**FIGURE 1:**
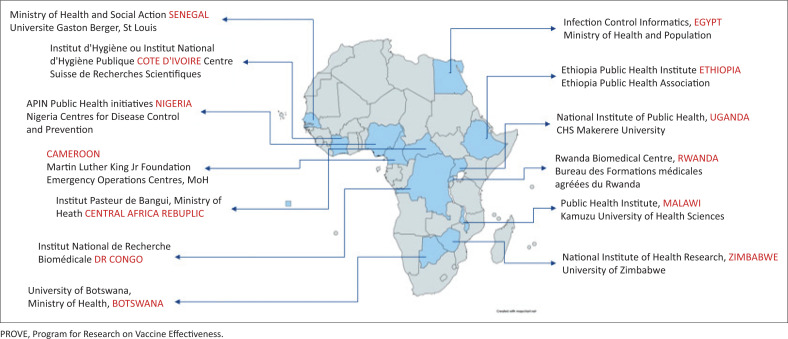
PROVE country presence.

As of early 2022, 13 member states were selected and prioritised for this research programme: Egypt, Senegal, Cote d’Ivoire, Nigeria, Ethiopia, Cameroon, Central African Republic, Democratic Republic of Congo, Uganda, Rwanda, Botswana, Malawi, and Zimbabwe (Figure 1).

### Data collection and management using the research electronic data capture (REDCap)

The programme opted to use REDCap to collect, manage, and store data in a secure environment. REDcap has many features designed for clinical and non-clinical research studies and has shown robust capabilities during previous surveys and clinical trials for COVID-19.^12^ The platform can also be operated in various languages, making it ideal for a multi-site programme where teams operate in different languages using similar data collection forms.

This strategy was hinged on the participating sites having collective data ownership, with Africa CDC hosting all the data for the subsequent harmonisation efforts. To enhance capacity, sites were engaged to appraise data management capabilities, identify local data managers, and provide customised training tailored to their experience with REDCap and anticipated platform access.

Although Africa CDC has vast experience in conducting health surveys and research, it had not used REDCap to collect and manage its research data. With support from the Infectious Diseases Institute, Makerere University, Africa CDC successfully obtained a licence to host REDCap for the contralised management of PROVE program data. This strategic licensing enabled the implementation of a neutral, centralised data management system across all PROVE in-country sites. To facilitate rapid scaling while ensuring standardisation, the data management team aligned the data collection tools with the generic protocol and data management plan. Corresponding datasets and data dictionaries were developed in both English and French, and site data managers received comprehensive training to effectively use the platform.

At the country level, sites made minor customisations to the PROVE REDCap tools to align with local needs and comply with in-country regulatory requirements, while preserving the programme’s vision of harmonised datasets.

### Data analysis strategy

Infectious Diseases Institute, Makerere University developed comprehensive data analysis plans for both the quantitative and qualitative components of the research protocol. These plans were tailored by the implementing country teams to align with their customised in-country research protocols. Adopting a collaborative approach, site statisticians and IDI data analysts worked together to prepare and review the datasets for analysis. Additionally, site teams were supported in conducting analysis using their preferred analysis software packages.

#### Data protection

As a co-custodian of the data generated from the programme in collaboration with AU member states, Africa CDC signed an overarching data-sharing agreement with IDI Makerere University. This agreement ensures the governance of data protection and ownership. Where local regulations required additional agreements, consortium partners – including IDI, the National Public Health Institute (or equivalent), and a local implementing research institution, entered into a tripartite agreement aligned with the overarching framework set by Africa CDC. Furthermore, study sites complied with in-country research data protection policies and adhered to Good Clinical Practice (GCP) guidelines.

All data access in REDCap was mandated by the site PIs through the site data managers, and access was restricted to each site through data access groups and password-protected accounts. This way, site teams could view, update, and edit only their site-specific data. However, the IDI and Africa CDC teams had overarching rights to access and view data for all sites. The data not collected through REDCap – such as key informant interviews and DHIS2 data - was uploaded by site data managers to a restricted-access online SharePoint in-country-specific folders.

#### Informed consent and confidentiality

All participants in human research must provide informed consent in the language of their choice, in accordance with the local ethics guidelines.^14^ In this research programme, informed consent was obtained from all participants where required. Participant data was anonymised by assigning identification numbers specific to each site, which were subsequently used to complete the Case Report Forms (CRFs) in REDCap. Consequently, all datasets utilised in the final analysis lacked participant identifiers.

### Cultural sensitivity and community engagement

In line with the guidelines of various African Union member states, local researchers adapted the research protocol to align with each country’s context while preserving the core research questions. This customisation process carefully considered scientific and operational aspects of data collection. It also refined questions and procedures, and addressed cultural nuances. Local ethics committees assessed and regulated these essential aspects. All proposed modifications were reviewed and either approved or addressed by the consortium leadership, with local investigators retaining final authority over protocol customisation decisions.

The research initiative benefited significantly from robust investment by the Risk Communication and Community Engagement (RCCE) arm of the Saving Lives and Livelihoods (SLL) programme. This investment increased community awareness around COVID-19 vaccines, securing key buy-in from local authorities and other stakeholders. Efforts by partner organisations supporting the RCCE were instrumental in disseminating information about interventions, creating a more receptive environment for the research teams.

Additionally, protocol training sessions served as an avenue for community engagement, with representatives from the local Ministry of Health and NPHI present. Their involvement lent credibility to the research programme. Moreover, queries raised by data collectors during these sessions created opportunities to brainstorm innovative strategies for reducing participant refusals and addressing cultural, political, and social factors that may have been overlooked during the research design development process.

### Programme monitoring strategies

The programme engaged in an iterative co-creation process to design a comprehensive monitoring and evaluation framework. This framework defined relevant and attainable performance indicators aligned with the objectives of successful research implementation in each country, ensuring findings and lessons were effectively communicated to local and consortium stakeholders for strategic planning and resource allocation.

The framework included bi-monthly engagement with in-country research teams to assess progress toward programme goals. The framework included bi-monthly engagement with in-country research teams to assess progress toward programme goals.

Additionally, weekly coordination meetings led by Africa CDC ensure seamless integration of research findings into the SLL initiative pillars. The monthly and quarterly reporting of key performance indicators occurred via the centralised DHIS2 digital platform hosted at Africa CDC which offered real-time progress assessments across the SLL initiative. Furthermore, leveraging REDCap for data collection significantly improved the programme’s monitoring capabilities, particularly in research data management. AFREhealth hosted a bi-weekly scientific meeting with the investigators to share best practices, appraise progress, and review operational data collection and analysis plans.

### Ethical considerations

**FIGURE 2:**
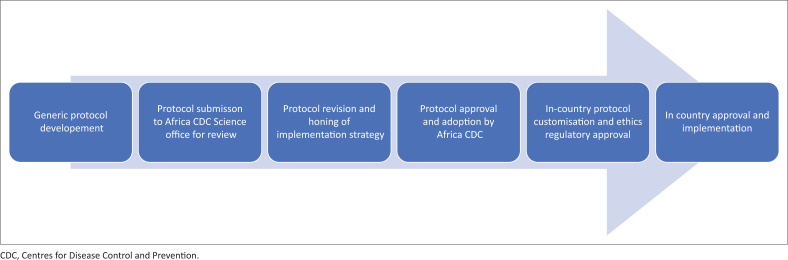
The research protocol development process.

Africa CDC and the consortium of implementing partners adopted a standardised research protocol implementation approach. This approach underwent a rigorous scientific review and was approved for implementation following the steps outlined in Figure 2. In-country protocol approvals were secured through local Ethics Review Boards and, where applicable, authorised by National Research Regulators. Establishing monitoring systems, therefore, requires adherence to all relevant requirements in order to maintain credibility and integrity.^13^

A generic monitoring plan was developed along with study-specific standard operating procedures, guided by the expertise of the IDI research department and regulatory monitoring team. These were shared with PIs at all the study sites for customisation and adoption. They served as references for daily study-related activities and procedures throughout the study period and astraining materials for site teams. Furthermore, where applicable, study sites have adopted and developed site-specific Standard Operating Procedures (SOPs) per their customised research protocols.

The strategy aimed to empower sites to establish their regulatory systems using either existing institutional frameworks or online share drives (e-binder). These e-binders functions as closed systems, accessible only to authorised individuals and the coordinating centre (IDI) for remote monitoring. PIs were tasked with designating points of contact – primarily study coordinators or data managers – who were responsible for uploading and maintaining essential documents in the regulatory file (e-binder). They also ensured proper oversight of all regulatory and ethical aspects of the programme. The IDI research monitoring team, in collaboration with site investigators, provided guidance and support throughout the process.

## Results

### Programme progress

The PROVE program successfully formed partnerships with research, health, and academic institutions in 13 member states, establishing impactful academia-Ministry of Health collaborations facilitated through NPHIs or their equivalents.

**FIGURE 3:**
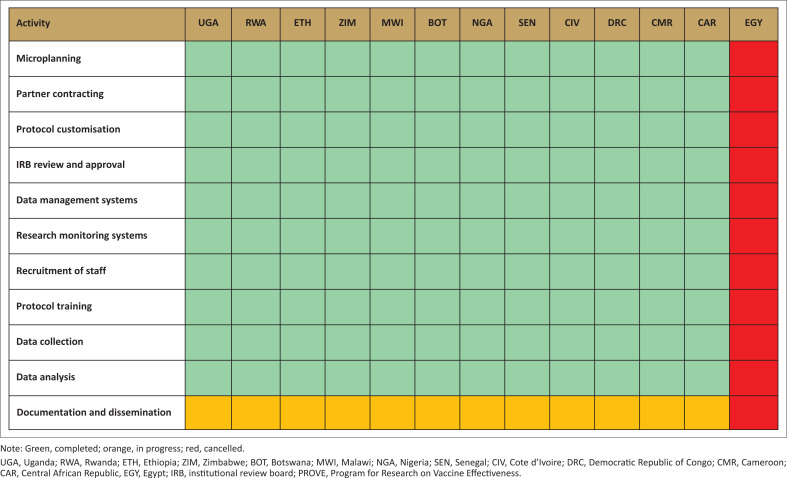
PROVE progress across the 13 AU member states.

Between May 2022 and June 2024, 12 AU member states were supported in adopting and customising the research protocol to align with their respective ethics and research guidelines. All 12 countries obtained ethical approval, trained research staff, and completed data collection activities. Moreover, all these countries remain dedicated to completing data analysis, results documentation, and dissemination for all the sub-studies (Figure 3).

Dissemination activities commenced, including two virtual Community of Practice (COP) events and two dissemination side events hosted at continental scientific conferences (the Conference on Public Health in Africa and the annual AFREhealth symposium). Preparations are in full swing for peer-reviewed articles in various journals and for dissemination at additional scientific meetings.

### Capacity building and training

Africa CDC and the consortium of implementing partners utilised structured training platforms and peer-to-peer interactions to facilitate knowledge exchange for research implementation and capacity building. One advantage is that the consortium uses a standardised research protocol, which helps ensure consistency in training materials and operating procedures across all research teams. To address the varying skill levels within the teams, peer-to-peer mentoring and the sharing of successful strategies were instrumental in facilitating horizontal skill transfer and ensuring standardisation across all consortium sites.

To build sustainable capacity for the NPHIs, the PROVE consortium established partnerships with the African Population and Health Research Center (APHRC) and the University of California, San Francisco. Together, they delivered two specialised training programmes in data science and implementation science, carefully tailored to align with the participants’ practice settings. These training sessions were conducted virtually and included weekly live sessions and self-paced tasks that participants completed alongside their regular work duties. The data science course spanned 12 weeks, while the implementation science course lasted eight weeks. Two cohorts were selected largely on the basis of the participants’ primary language of instruction for each training session. In June 2024, 60 and 61 participants had completed the data science and implementation science courses, respectively.

### Challenges and mitigation strategies

For several reasons, in-country ethics review committees frequently raised questions about the customisation of the research protocol within their respective countries.

A primary concern was the shifting of national priorities. Several partner countries questioned the timeliness and relevance of the PROVE study’s objectives, given the evolving epidemic and shifting national priorities. The emergence of the Omicron variant resulted in a surge in COVID-19 cases during the protocol development phase. However, the surge diminished once the protocol customisation and approval processes were finalised. Consequently, some ethics committees expressed reservations about the pertinence of the vaccine effectiveness studies. However, it is worth noting that most countries still prioritise increasing vaccination coverage. Ongoing engagement and clear communication on the importance of aggregated estimates in generating the first-ever African evidence of COVID-19 vaccine effectiveness were well-received.

Additionally, the secondary objectives concerning vaccine uptake dynamics among African healthcare workers were considered essential for shaping strategies to enhance vaccine acceptance among frontline professionals. This experience highlighted the diversity of research priorities, practices, as well as and governance frameworks crucial for the success of any research project. Furthermore, it reinforced the importance of democratising public health in Africa.

Another area of concern was scepticism surrounding novel study designs, particularly the test-negative study design (TND),^15^ which had been rarely used in implementation research. Several ethics committees questioned the rationale, appropriateness, and prior use of the test-negative case-control design proposed for evaluating vaccine effectiveness. To address these concerns, the PROVE team engaged with the respective scientists, providing guidance and responding to queries from the ethics teams. As a result, countries could prove and implement the study activities. The team also presented evidence^15^ of the successful use of the test-negative design (TND) in evaluating COVID-19 vaccine effectiveness in other settings.

Changes in the eligibility criteria of the study population were a notable issue, partly due to evolving international guidelines for vaccination. The definitions of adults and emancipated adults varied across countries, necessitating harmonisation when establishing eligibility criteria. The approval of COVID-19 vaccines for children in select countries prompted government policy adjustments to align with the expanded eligibility for COVID-19 vaccination. The PROVE generic protocol initially focused on evaluating effectiveness among adults, given the ease of obtaining consent and the high vaccination rates in this demographic. However, during the protocol customisation process, member states that had commenced vaccinating children were allowed to enrol participants below the consent age, provided parental assent was obtained.

Authorship in collaborative projects poses significant challenges, including determining contributions, deciding the order of authors, resolving disputes, navigating institutional and cultural differences, maintaining ethical standards, and balancing credit with accountability.

Achieving equitable collaboration and authorship within PROVE was underpinned by several strategic measures. The programme’s governance structure, overseen by NPHIs, ensured a balanced and inclusive leadership approach. To further enhance equity in authorship, the programme developed a dissemination and authorship plan firmly rooted in the International Committee of Medical Journal Editors (ICMJE) authorship guidelines. This plan incorporates rigorous internal control processes that engage all stakeholders and their roles (Table 2), thus safeguarding fair attribution of contributions. Additionally, by actively integrating with the administrative structures of local research institutions, the programme promoted autonomy and ownership among participating entities, fostering a conducive environment for equitable research partnerships and authorship recognition. These efforts underscored the programme’s commitment to ensuring that research outcomes and authorship are grounded in fairness and inclusivity.

**TABLE 2:** Collaboration and authorship roles for partners.

Stakeholder entity	Roles and responsibilities
Africa CDC and Mastercard Foundation	Overall oversight and coordination of all aspects of the programme serve as the ultimate stewards of the information generated from the programme.
Ministry of Health and National Public Health Institutes	Oversee programme activities at the country level, including collecting, managing, disseminating, and safeguarding the valuable research findings from PROVE.
In-country partner institutions	Serve as bridges between the Ministries of Health and the Infectious Diseases Institute by facilitating communication and coordination with international stakeholders and promoting knowledge exchange.
Infectious Diseases Institute, Makerere University (IDI)	Is the lead partner for the Implementation Science Pillar within the SLL initiative and responsible for designing, implementing, and managing all research activities under PROVE.
Africa Forum for Research and Education in Health (AFREhealth)	Provides support for continental collaboration under the implementation science pillar by engaging in knowledge-sharing platforms and diverse stakeholder engagement activities to enhance the programme’s impact.

CDC, Centres for Disease Control and Prevention; PROVE, Program for Research on Vaccine Effectiveness; SLL, Saving lives and livelihoods.

The programme brought together diverse stakeholders – NPHIs, senior academics and policymakers from 12 Member States – many working together for the first time. Building trust, aligning priorities, and navigating differing communication styles and institutional cultures was a challenge in the early stages of setting up the programme.

Team dynamics were enhanced through joint celebration of early wins including approval of protocols and using opportunities such as protocol training workshops for team-building activities. Team specific conflict resolution and arbitration measures were undertaken where the programme leadership deemed the conflict to be of high risks to the delivery of the programme. Despite initial disparities in workflows and experience levels, these efforts fostered collaboration, mutual respect, and alignment of goals.

### Prospects and sustainability

#### Planned scientific publications and dissemination

With the guidance of Africa CDC’s science office and the SLL leadership, IDI developed a dissemination plan to ensure the effective communication of research findings, outcomes and recommendations to relevant stakeholders, including policymakers, healthcare practitioners, public health agencies, and others in Africa.

Site investigators in each implementing country are documenting study results for each sub-study in accordance with the customised protocols to generate research findings. These will be published on several platforms, such as scientific conferences, communities of practice, dissemination meetings with MoH stakeholders, and peer-reviewed journals. The IDI team will compile and document all country-specific results into a comprehensive report for each sub-study.

#### Policy influences: Advocacy and strengthening local health systems

The collaboration between NPHIs and local academic and research institutions integrate policymakers into the research process, bridging the gap between knowledge generation and its practical application to influence policy. This partnership n will also foster a shared understanding of the challenges involved in conducting research within specific country contexts, creating an opportunity to advocate for policies that address e barriers to the rapid execution of research during public health emergencies.

#### Building sustainable research infrastructure

The PROVE program employs a multifaceted approach centred on standardising research administration, ethics review frameworks, and establishing robust systems for partnership and collaboration. Capacity-building efforts focus on institutional governance, context-specific compliance audits, data standardisation and workforce development, especially for young scientists.

The adoption of standardised ethics review frameworks served as a foundational pillar. These frameworks ensure that research adheres to the highest ethical standards and follows streamlined processes by providing universal guidelines. This fosters trust among stakeholders and minimises redundancy in research efforts, thereby optimising resource allocation and reducing ethical ambiguities.

Institutional governance structures bolster transparency, accountability, and coordinated resource allocation, aligning research priorities with societal needs. Context-specific compliance audits that are conducted independently reinforce ethical compliance, ensuring research projects conform to established guidelines and open opportunities for international collaboration.

The PROVE program also plans to build on the shared resources for data standardisation and harmonisation, facilitating seamless collaboration among researchers from diverse institutions and enhancing the quality and comparability of research data.

Prioritising the development of robust institutions over the reliance on individual principal investigators is crucial for long-term sustainability. Robust institutions persist beyond individual researchers, guaranteeing the continuity of research initiatives. This approach cultivates a collaborative culture and mitigates the risk of research dependency on a select few. Adopting these principles establishes a resilient research ecosystem capable of making lasting contributions to society.

#### Scale up and expansion

WHO’s reclassification of COVID-19 in May 2023 and the rise of its global significance marked a pivotal milestone for the SLL programme. Responding to this shift, Africa CDC and the Mastercard Foundation recalibrated the SLL initiative to safeguard vulnerable populations, integrating COVID-19 response activities into mainstream healthcare systems in AU member states. In this evolving landscape, the implementation science pillar took on greater prominence, strategically leveraging data from healthcare worker vaccine uptake surveys to inform policy recommendations and support the integration of COVID-19 vaccination into national vaccination programmes across member states. This updated strategy by the SLL initiative seamlessly integrates with the existing research platform while broadening its scope to include sustainability initiatives, such as creating an academic consortium for health security in Africa. Additionally, the PROVE consortium reaffirms its commitment to strengthening capacity-building efforts for the National Institutes of Public Health (NIPHs), thereby bolstering health security across the continent.

#### Potential for replication in other regions

The programme demonstrate significant potential for replicating its implementation model, driven by several factors that highlight its robust and adaptable framework. Firstly, its presence across all five regions of the African Union positions it as a comprehensive and continent-wide initiative, fostering opportunities for knowledge transfer and cross-regional replication. Secondly, the programme’s leadership by local institutions, aligns with the Africa CDC’s strategy to strengthen public health systems. This reflects a commitment to sustainability and local capacity building, thus laying a solid foundation for replication. Secondly, the programme’s leadership by local institutions, in line with the Africa CDC’s strategy to strengthen public health systems, reflects a commitment to sustainability and local capacity building, providing a solid foundation for replication.

Furthermore, the programme’s reliance on a generic research protocol characterised by well-defined objectives, standardised methods, and meticulous implementation plans enhances its replicability. This approach ensures that research endeavours across diverse contexts can adhere to a structured framework while accommodating region-specific nuances. Additionally, the utilisation of standardised data collection and management platforms, notably the REDCap system, streamlines data processes and promotes uniformity, facilitating replication across multiple settings.

Workforce development is a pivotal element of the programme’s implementation model. The initiative invests in enduring human capital development by training research teams and equipping them with specialised data management and implementation science skills. This approach strengthens the immediate research efforts and fosters the transfer of knowledge and expertise that can be utilised beyond the programme’s scope.

The programme’s commitment to comprehensively documenting lessons learned is instrumental in ensuring its replicability. By capturing insights, challenges and best practices, the programme creates a repository of knowledge that can guide future initiatives and facilitate peer learning. This approach not only enhances the programme’s effectiveness but also benefits the broader academic and public health community by sharing valuable experiences and insights.

## Discussion and conclusion

PROVE’s success lies in its ability to unite policymakers and academia to generate evidence and foster international collaboration, delivering impactful outcomes within local contexts. This localised approach is a cornerstone of implementation science and underscores the importance of context in adopting and sustaining new practices despite diverse research frameworks and governance structures.^16^ Africa CDC can play a pivotal role in spearheading the development of a unified research strategy among AU member states, leveraging the generic research protocols approach as a scalable concept for such initiatives. The diversity of in-country research governance frameworks is a significant consideration in the design of multi-country research projects. Respecting these frameworks is vital, but they can sometimes lead to protocol modifications that compromise the comparability of outcomes and the standardisation of research procedures across countries. Balancing adaptability to local contexts with the need for uniformity remains an ongoing challenge in multi-country research collaborations.

PROVE’s iterative monitoring, evaluation and feedback process allowed continuous improvement. This adaptive methodology ensured that interventions remained responsive to emerging challenges and opportunities. The SLL initiative has committed to supporting the integration of COVID-19 service delivery frameworks into routine healthcare, thereby strengthening the continent’s preparedness for future pandemics while ensuring vaccines reach the most vulnerable populations in Africa.^17^ As the programme enters the critical phase of integrating the evidence generated into national programming, this commitment provides an excellent opportunity to evaluate the impact of PROVE’s recommendations on sustaining collaborations and advancing the goals of Africa’s New Public Health Order.

The COVID-19 pandemic, as a universal challenge for all member states, offered a unique situation for developing an applicable and inclusive research agenda. However, there is still more to learn as individual member states’ health research priorities take centre stage, especially since the pandemic was declared an ongoing health concern. Africa CDC will have a more significant role in mapping the gaps in continental health security and setting a continental research agenda.

To position human capital for the effective implementation of Africa’s New Public Health Order, PROVE has invested in training and ongoing education for participating NPHIs. These efforts focus on implementation and data science, strengthening local data availability to support l collaborative evidence-generation efforts at both national and international levels. The PROVE consortium will archive the developed content and curricula at Africa CDC, ensuring its preservation and accessibility for other AU member states that were not primary beneficiaries of the programme.

We acknowledge the limitations in this initiative concerning gender parity considerations, particularly at the institutional level among decision-makers and policymakers. While our study provides critical foundational insights, it did not explicitly explore the role of gender inclusion within these institutions’ decision-making processes. We recognise the importance of integrating gender parity considerations in all aspects of research and health policy, as extensively established in literature.^18,19^ Layering a gender-inclusive framework on this collaboration approach will enrich the broader applicability and equity of our findings.

In conclusion, multi-country research collaborations in Africa hold immense promises for addressing research priorities and tackling global health challenges. However, they require careful navigation of diverse national research landscapes and priorities. By adopting an implementation science approach, a strong foundation has been established for effective coordination, capacity building, and operational excellence, all of which are essential for addressing critical global health issues on the continent.
